# Humanitarian Mission in Pediatric Cardiothoracic Surgery: A Recipient's Perspective

**DOI:** 10.3389/fped.2019.00230

**Published:** 2019-06-07

**Authors:** Mohd Rizal Mohd Zain, Ahmad Mahir Shamsuddin, Ahmad Zuhdi Mamat, Ariffin Marzuki Mokhtar, Saedah Ali, Yen Chuan Chen, Antonio F. Corno

**Affiliations:** ^1^Department of Pediatrics, School of Medical Sciences, Universiti Sains Malaysia, Kubang Kerian, Malaysia; ^2^Hospital Universiti Sains Malaysia, Health Campus, Universiti Sains Malaysia, Kubang Kerian, Malaysia; ^3^Cardiothoracic Unit, Department of Surgery, School of Medical Sciences, Universiti Sains Malaysia, Kubang Kerian, Malaysia; ^4^Department of Anesthesia, School of Medical Sciences, Universiti Sains Malaysia, Kubang Kerian, Malaysia; ^5^Cardiothoracic Surgery Department, Sabah Heart Centre, Queen Elizabeth Hospital II, Kota Kinabalu, Malaysia; ^6^East Midlands Congenital Heart Center, Glenfield Hospital, Leicester, United Kingdom; ^7^Cardiovascular Research Center, University of Leicester, Leicester, United Kingdom

**Keywords:** surgical mission, congenital heart defects, volunteerism, charity, surgical outcomes

## Abstract

**Introduction:** Pediatric cardiac surgical mission programs are deemed as common practice, especially in developing nations funded by international non-governmental organizations (NGOs). This article presents and discusses the results and strategies implemented by this partnership, aiming at achieving the autonomy of the local center by this collaboration.

**Materials and Methods:** A retrospective review was conducted on patients with congenital heart disease who underwent surgical intervention from the beginning of the NGO collaboration (September 2015) until November 2018 in an existing cardiac center. In between those visits, any congenital heart disease patient with Risk Adjustment Congenital Heart Surgery (RACHS)-1 Category 1–3 would be discussed in a local multi-disciplinary meeting with regards to the feasibility of the surgery being performed by the local members.

**Results:** A total of 60 operations were performed during the trips. Throughout the visit, 46% (28) of the operations were performed by the local surgeon, with or without assistance from the visiting surgeon. Between September 2015 and November 2018, 27 cases were also performed by the local team independently. For the 27 cases performed by the local team independently, the median age of the patient was 42 days (ranging from 14 days to 20 years old), with median body weight of 3.2 kg (ranging from 2.8 to 64 kg).

**Conclusion:** Humanitarian pediatric cardiac surgical missions are safe to be done for the population in need. In order to achieve autonomy, continuous efforts by both teams are crucial, as the cooperation by the two parties ensures that the objectives are achieved.

## Introduction

Congenital Heart Disease (CHD) occurs in 1% of births per year (1). The detection rate and management to encounter CHD are increasing trends, especially in developed countries. In least developed countries, CHD is still a major burden for the population and most of children are left unattended. According to the WHO, a cardiac center with 300–500 operations performed annually is required in order to accommodate a population of 2 million people (2). Nonetheless, there is not even a single pediatric cardiac center within areas of population of 15–70 million people in certain parts of developing countries (3). This also happens in Asia, where on average there is only one pediatric cardiac center for 16 million population (4). To tackle the issue, transferring, and accepting the expertise from developed countries by having a regular surgical visit to the areas that mostly in need are practiced. It might be asserted that the cost of sending a cardiac surgical team to operate on 10–20 children in the selected country is equal to sending one abroad (5, 6). Perhaps Pezzella (7) best elaborated this when he wrote “the era of bringing patients to the United States for free cardiac surgery is over.”

As such, cardiac surgery services are limited in most developing countries, and many patients have no choice but to live in morbid conditions. In contrast, there are several cardiac programs available in other regions, but there are only a few caseloads noted. It is commonly practiced by health providers in developing nations to run collaborative mission programs sponsored by international or domestic non-governmental organizations (NGOs). The aim of this collaboration is to develop local cardiac surgery institutions to provide continuous treatment for patients in developing countries. Numerous approaches have been used: regionalization, “safari” missions, twinning programs, and “travels of hope” (8, 9). Since then, many non-governmental humanitarian organizations (NGOs) have been initiating pediatric cardiac surgery services in developing countries. This article presents and discusses the results and strategies implemented by this partnership, aiming at achieving autonomy at the local centers by collaborating with the NGO.

## Materials and Methods

A retrospective review was conducted on patients with congenital heart disease who underwent surgical intervention from the beginning of the NGO collaboration (September 2015) until November 2018 in an existing cardiac center. It is a part of the result in our internal clinical audit data regarding surgical humanitarian mission in the local center. This study has been revised by the local ethical committee [Human Research Ethics Committee USM (HREC)] and was exempted from ethical review. No informed consent was taken because it is a retrospective study.

The local unit's collaboration with the NGO for pediatric cardiac surgical mission was established in September 2015 with one of the co-authors (AFC). Since then, there have been 6 visits by the U.K. teams until November 2018. In general, the visiting team usually consists of 7–8 people, including a consultant pediatric cardiac surgeon, consultant intensivist, consultant pediatric cardiologist, Pediatric Cardiac Intensive Care Unit (PCICU) Registrar, and 1 or 2 PCICU nurses. In only one of the visits were there an operating room nurse and a consultant anesthetist.

The local cardiothoracic unit was established in 2001, and a majority of the cases performed were adult cardiothoracic cases. The pediatric cardiac surgical unit was established in 2013 but later in early 2015, it was closed due to the migration of the pediatric cardiac surgeon who contributed to organizing the unit from the beginning. The center received nearly 50 cases of pediatric congenital heart disease per year requiring surgery, and all of them were sent to the nearest pediatric cardiac center, which was 600 km away with very difficult transfer because of the geographic situation. The local team was composed of two fully trained adult cardiac surgeons (one of them had special interest in pediatric congenital heart cases), two cardiac anesthetists, one pediatric cardiologist, three neonatologists, and a few fully trained supporting staff (perfusionist, PCICU nurses, operating room nurses, respiratory physiotherapist, etc.) in the adult cardiothoracic program. There were only 3 cardiac Intensive Care Unit (ICU) beds available, which allowed for ~90–120 cases per year.

After all the visits by the U.K. teams, all the patients were managed under a local multi-disciplinary approach, and there was also direct communication with the lead visiting team that had left. After the visit, any congenital heart disease patient with Risk Adjustment Congenital Heart Surgery (RACHS)-1 Category 1–3 would be discussed in a local multi-disciplinary meeting with regards to the feasibility of doing surgery by the local members. Expert opinions from the visiting team might be consulted for further evaluation. This involves frequent exchange of e-mail and the use of social media, e.g., WhatsApp. This autonomy led the local cardiac surgeon to choose the cases and proceed to perform the operations with the support of the local expertise available.

## Results

From September 2015 to May 2018 there were a total of six visits from U.K. teams to the local hospital, 60 patients were identified for surgical treatment, and a total of 60 operations were performed. For the 60 cases performed during missions, the median age of the patient was 2 years old (ranging from 15 days to 30 years), with a median body weight of 11 kg (ranging from 2.7 to 53 kg) and median height of 85 cm (ranging from 45 to 159 cm). Forty-seven (78%) of them were on-pump cases, while 13 (22%) were off-pump cases. During the visit, 46% (28) of the operations were performed by the local surgeon, with or without assistance from the visiting surgeon. The procedures done included 17 VSD closures, 3 ASD closures, 5 Pulmonary artery bandings, 2 AVSD repairs, and 1 TOF Repair.

From September 2015 to November 2018, there had already been 27 cases performed by the local team independently. The complexity of the case ranged from Modified Black-Taussig Shunt in neonates, VSD closure and PDA ligation (Table 1). For the 27 cases performed by the local team independently, the median age of patients was 42 days (ranging from 14 days to 20 years), with median body weight of 3.2 kg (ranging from 2.8 to 64 kg). Seven (26%) of them were on-pump cases while 20 (74%) were off-pump cases.

**Table 1:** List of surgical procedures performed by local teams independently.

**Number of procedures**	**Surgical procedures**	**Additional procedures**
7	PDA closure	
6	VSD closure	3 RVOT reconstruction
4	Modified Blalock-Taussig Shunt in neonate	1 Carotid Artery-Main Pulmonary Artery Anastomosis
4	Permanent Pacemaker Implantation	1 Epicardial pacing wire insertion1 Pacemaker-box change
3	Pulmonary Artery Banding	1 PDA closure
2	CCAM Excision	
1	Removal of dislodged PDA Device under CPB	1 RA wall tear repair

*CCAM, Congenital Cystic Adenomatoid Malformation; CPB, cardiopulmonary bypass; PDA, Patent Ductus Arteriosus; RVOT, Right Ventricular Outflow Tract; VSD, Ventricular Septal Defect*.

Post-operatively, all the patients were managed by a multi-disciplinary team, which included a pediatric cardiologist, neonatologist, cardiac surgeon, and anesthetist. Out of 27 cases performed by the local team, 3 (11%) of the patients died post-operatively. Two-thirds of the patients were neonates with RACHS-1 Risk Category 3, with the body weight ranging from 2.90 to 3.50 kg, and the mortality occurred within 4–8 h post-operatively. The first patient died due to severe heart failure secondary to pulmonary overcirculation after neonatal Blalock-Taussig shunt for severe PS with underlying cCTGA. The second mortality was after pulmonary artery banding in a patient with Down syndrome and complete atrioventricular septal defect. The third mortality was in a patient with a dislodged Patent Ductus Arterious (PDA) occluder device during transcatheter procedure, complicated with cardiac tamponade requiring cardiopulmonary resuscitation x 3 preoperatively. Urgent operation was done under cardiopulmonary bypass for PDA device retrieval.

## Discussions

“*The success should not be measured by the number of successful operations of any given mission, but by the successful operations that our colleagues perform after we leave”*.

–Dr. Gary Raff (10)

Dearani et al. (8) also mentioned the complexity of the cases that could be achieved in an NGO collaboration with the existing cardiothoracic services, which could be categorized from Level 1 to Level 4 in complexity. The role of the visiting team as a long-term, off-site collaborator was also important in accomplishing autonomy. To achieve this, we adopted the “twinning program” (8) approach, in which the local surgeon was attached to an established surgeon in a center of excellence. As for the local surgeons, performing those surgeries with the visiting team helped them to be familiar with the surgical procedures. In our case, the local surgeon performed at least 48% of the cases during the visit. This could only be achieved with careful selection of the cases and interactive communication between both the local and visiting surgeons. After the visit, all the patients had been managed by local multi-disciplinary approaches, with the assistance of direct communication with the lead visiting team. This constant way of working had developed the local team's confidence in handling pediatric cardiac patients, which in hand also led to autonomous development of the team.

We believe that the success of the program in achieving autonomy was based on both interdisciplinary communications as well as good insight of the local team regarding our strength and weaknesses. The selection of cases includes RACHS-1 Category 1–3, with a bodyweight of more than 5 kg for on-pump cases and avoidance of on-pump cases on neonates if permissible (8). Apart from that, minimizing ICU length of stay and early extubation were the main objectives postoperatively. This was due to the limitation of resources and trained personnel in the PCICU. Accidental extubation with frequent reintubation and errors in preparation of medications seem to be the most common errors found in prolonged mechanical ventilation or ICU stays. Whenever possible, one-to-one model pairing between the local team and visiting team members, as well as same-site recurring voluntary visits, should be done in order to properly assess the development of the local team achievement (11).

With regard to the 27 cases operated on our own (without the involvement of the visiting team), we had a relatively high mortality rate. In this notion, we must agree that most of the mortality occurred in very high-risk cases, with a high level of complexity and involving “rescue surgery.” However, this had not stopped completely the activities and led us to better select cases and improve our care to boost surgical activities. In order to improve the clinical practice, the mortality and morbidity meetings were conducted by the local team together with the foreign team during the visits.

### Sustainability

From a financial standpoint, the survival of an NGO collaboration with local cardiac centers depends on fulfilling the needs of the community by delivering quality care and publicizing its success. Sometimes, the visiting team's accommodation, hospitality, and transportation might be needed and the funds may come from the contribution of local communities rather than from the visiting NGO itself. Occasionally, the patients also need support in term of hospital bills and intervention involved (e.g., usage of tissue valve may not be subsidized by most hospitals). The program should be advertised in the local media, press, and online to increase public awareness, as well as to increase funding for the local team (5).

Data collection and analysis was a key for sustainability of this program. The data should involve patient demographics, diagnosis, surgical procedures, mortality, morbidity, complexity-adjusted outcome analysis, and resources involved, which could demonstrate the program's growth and its impact. Some surgical procedures could be modified more locally based with the resources available to perform the surgery. For example, doing five arterial switches in a 1-week NGO visit may overwhelm the resources of the local hospital, and it is less effective in training the local surgeon as well. These factors must be considered when determining whether a procedure could be supported and sustained in a resource-limited hospital or not (12). Additionally, developing pediatric specialty services might require longer commitment, often exceeding 5 years before a surgical center reaches its independence (6).

### Problems and Risks

Safety might be compromised during the visit since the local teams are not familiar with the large number of cases in a short trip, and this will also affect other specialties that use the same facilities (3). Another consideration that needs to be taken for a local team is the preparedness of the local setting in accepting the visiting NGO. The preparation might include hospitality, operating theater setting, equipment, and disruption of regular schedule. For example, other operation theaters might need to be closed during the visit to better accommodate the whole visiting surgical activities. The visiting team should also be prepared for any unsafe or substandard environments. For instance, when the ICU/OT are under renovation this might cause power supply disruption.

Interpersonal disagreement within the institution, political appointments without ability to expand the collaboration, and behavioral factors are some of the reasons that can lead to failure of this partnership. Occasionally, lack of hospital support from the administrators is an unavoidable problem (6). In our case, we were lucky because the administration was fully supportive of the program. This was achieved by continuous discussion and communication with all the parties involved. Sometimes, the lack of continuous communication between the local team and visiting team after the visit might cause significant distance in helping the local team to develop. As such, the majority of visiting surgeons do not maintain the communication with the local surgeon. This must be tackled, and the continuous communication should be extended to all levels of medical practitioner, not being limiting to surgeons only.

## Conclusions

Humanitarian pediatric cardiac surgical missions are safe to be performed on the population in need. Achieving autonomy is possible with continuous effort provided by both teams. Cooperation between both teams will ensure all the objectives can be achieved.

## Author Contributions

AS, MM, and AZM contributed to the conception and design of this study. AMM and SA wrote sections of the manuscript. AC and YC revised the manuscript critically and contributed to the interpretation of data. All authors listed have made a considerable and direct contribution to the work, and approved it for publication.

### Conflict of Interest Statement

The authors declare that the research was conducted in the absence of any commercial or financial relationships that could be construed as a potential conflict of interest.
